# Adjusted green spectrophotometric determination of favipiravir and remdesivir in pharmaceutical form and spiked human plasma sample using different chemometric supported models

**DOI:** 10.1186/s13065-023-01001-5

**Published:** 2023-07-27

**Authors:** Mohamed S. Imam, Ahmed H. Abdelazim, Sherif Ramzy, Afnan S. Batubara, Mohammed Gamal, Safwan Abdelhafiz, Abdallah M. Zeid

**Affiliations:** 1 Pharmacy Practice Department, College of Pharmacy, Shaqra University, Shaqra, 11961 Saudi Arabia; 2 Clinical Pharmacy Department, National Cancer Institute, Cairo University, Fom El Khalig Square, Kasr Al-Aini Street, Cairo, 11796 Egypt; 3 Pharmaceutical Analytical Chemistry Department, Faculty of Pharmacy, Al-Azhar University, Nasr City, Cairo, 11751 Egypt; 4 Department of Pharmaceutical Chemistry, College of Pharmacy, Umm Al-Qura University, Makkah, 21955 Saudi Arabia; 5 Pharmaceutical Analytical Chemistry Department, Faculty of Pharmacy, Beni-Suef University, Beni-Suef, 62514 Egypt; 6 Faculty of Pharmacy, Al-Azhar University, Cairo, 11751 Egypt; 7 Department of Pharmaceutical Analytical Chemistry, Faculty of Pharmacy, Mansoura University, Mansoura, 35516 Egypt

**Keywords:** Favipiravir, Remdesivir, CLS, PCR, PLS

## Abstract

The environmentally friendly design of analytical methods is gaining interest in pharmaceutical analysis to reduce hazardous environmental impacts and improve safety and health conditions for analysts. The adaptation and integration of chemometrics in the development of environmentally friendly analytical methods is strongly recommended in the hope of promising benefits. Favipiravir and remdesivir have been included in the COVID-19 treatment guidelines panel of several countries. The main objective of this work is to develop green, tuned spectrophotometric methods based on chemometric based models for the determination of favipiravir and remdesivir in spiked human plasma. The UV absorption spectra of favipiravir and remdesivir has shown overlap to some extent, making simultaneous determination difficult. Three advanced chemometric models, classical least squares, principal component regression, and partial least squares, have been developed to provide resolution and spectrophotometric determination of the drugs under study. A five-level, two-factor experimental design has been used to create the described models. The spectrally recorded data of favipiravir and remdesivir has been reviewed. The noise region has been neglected as it has a negative impact on the significant data. On the other hand, the other spectral data provided relevant information about the investigated drugs. A comprehensive evaluation and interpretation of the results of the described models and a statistical comparison with accepted values have been considered. The proposed models have been successfully applied to the spectrophotometric determination of favipiravir and remdesivir in pharmaceutical form spiked human plasma. In addition, the environmental friendliness of the described models was evaluated using the analytical eco-scale, the green analytical procedure index and the AGREE evaluation method. The results showed the compliance of the described models with the environmental characteristics.

## Introduction

 The environmentally friendly design of analytical methods is gaining interest in pharmaceutical analysis to reduce hazardous environmental impacts and improve safety and health conditions for analysts. Application of chemometrics in green methods development is still limited. The adaptation and integration of chemometrics in green analytical method development is highly recommended with hoping for promising benefits [1, 2]. In general, chemometric tools provide feasibility to decrease data dimensionality and assign the irrelevant variables or objects as well as eliminate the irrelevant variables. Furthermore, chemometrics introduce unique possibility to whole data set treating as a group of nonrelated variables of objects [3]. Chemometrics can be applied in analytical procedure development targeting materials and energy savings and the minimization of the procedure’s environmental impact that directly refer to the procedure’s greenness [4].

**Fig. 1:**
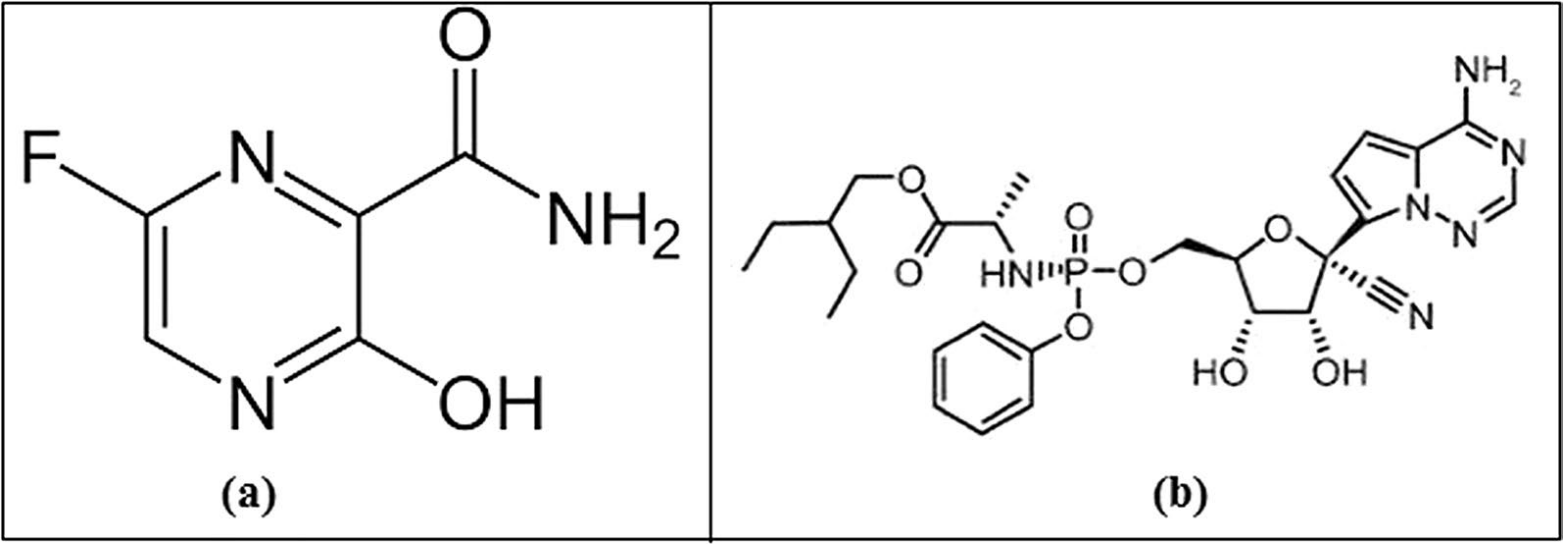
Structural formula of FV (**a**) and RV (**b**)

Favipiravir [FV], Fig. 1a, is a purine nucleic acid analog with known antiviral activity considered in many infections. FV is activated to the phosphorribosylated and delivers activity via inhibition of RNA-dependent RNA polymerase [5]. Numerous clinical studies have demonstrated the effectiveness of FV in the treatment of COVID-19 [6, 7]. Previous research has described some analytical methods for the determination of FV, including HPLC methods [8, 9] and electrochemical method [10].

Remdesivir [RV], Fig. 1b, is a nucleotide prodrug of an adenosine analog. RV exerts its activity by binding to viral RNA-dependent RNA polymerase and subsequently inhibiting viral replication by premature termination of RNA transcription. RV has confirmed activity against COVID-19. The FDA has approved the use of RV for the treatment of COVID-19 in hospitalized adult and pediatric patients [11–13]. Previous research has described some analytical methods for the determination of RV, including HPLC methods [14, 15] and spectrophotometric methods [15, 16].

In fact, FV and RV were listed in the COVID-19 treatment guidelines of several countries. They were prescribed simultaneously for COVID-19, so it is highly recommended to develop an analytical method that allows the determination of FV and RV in biological samples. Few analytical methods were developed for determination of FV and RV in their binary mixture including TLC-densitometric method [17] and fluorescence-based analytical method [18].

In this article, adopted spectrophotometric determination of FV and RV was developed and applied for pharmaceutical form and spiked human plasma using several chemometrically assisted models. Three advanced chemometric models, the classical least squares [CLS], principal component regression [PCR], and partial least squares [PLS] methods [19, 20] were constructed and optimized for the integrated green spectrophotometric determination of the studied drugs. Greenness evaluation of the described models was done using different tools, the analytical eco-scale [21], the green analytical procedure index [22] and the AGREE evaluation method [23]. The described models showed superiority and agreement with the greenness characteristics in terms of the common green metric values. The authors hope to provide a promising challenge for achieving green goals by integrating chemometric tools and applying them with green assessment metrics.

## Experimental

### Materials and solvents

Pure powders of FV (99.10%), RDV (99.62%) were supplied by Benchmark Health Company, Cairo, Egypt. Drug-free human plasma of healthy volunteers was supplied by Blood Bank, Al-Azhar University Hospital, Damietta, Egypt. Remdesivir® vials [100 mg RV] and Anviziram® tablets [200 mg FV] were kindly purchased from local market. Ethanol, was procured from Sigma-Aldrich, Germany.

### Apparatus and software

Shimadzu UV-Visible 1650 Spectrophotometer, Tokyo, Japan. UV-Probe personal spectroscopy software version 2.1. (Shimadzu). PLS model was implemented in Matlab R2013b (8.2.0.701) and manipulated by PLS toolbox software version 2.1, was accessed by Umm Al-Qura University, Makkah, Saudi Arabia.

### Standard solutions

Standard stock solutions of FV (100 µg/mL) and RD (100 µg/mL) was prepared by dissolving 10 mg of each pure powder in 50 mL of ethanol, and the volume was made up to 100 mL with ethanol.

### Procedures

#### CLS, PCR and PLS models design

Calibration and validation sets were created using the common approach, five-level two factorial design. Twenty-five mixtures of FV and RD were initially constructed and divided into two defined sets, calibration and validation sets. These mixtures were prepared by transferring different volumes of FV and RD from the standard stock solutions (100 µg/mL) into 10 mL volumetric flasks. The prepared solutions were adjusted to the mark with ethanol. The absorbance spectra of the prepared samples were scanned over the wavelength range 220–400 nm with an interval of 1 nm. CLS, PCR and PLS models were processed by transferring the spectral data into Matlab R2013b (8.2.0.701). Thirteen samples were used as calibration set and twelve samples were used as validation set. The predictability of the CLS, PCR and PLS was evaluated. In general, all described procedures were performed in accordance with relevant guidelines and regulations.

#### Procedures for the determination of the pharmaceutical dosage forms

Ten Anviziram® tablets were scratched, weighed, and finely powdered. An appropriate weight of powder corresponding to one tablet was placed in a 100 mL volumetric flask and the volume was made up to 50 mL with ethanol. The solution was shaken vigorously for 30 min and filtered. The volume was made up to the mark with ethanol to prepare a stock solution containing 2 mg/mL FV. Different concentrations were prepared and determined regarding to the procedures of the mentioned models.

Five Remdesivir® vials were weighed and an equivalent weight to one vial was transferred to a 100-mL volumetric flask and the volume made up to 50 mL with ethanol. The solution was shaken for 30 min and filtered. The volume was completed to 100 mL with ethanol to prepare a stock solution containing 1 mg/mL RV. Different concentrations were prepared and determined regarding to the procedures of the mentioned models.

#### Procedure for determination of FV and RD in spiked human plasma

Different spiked human plasma samples, considering that the mean plasma Cmax for FV (4.43 µg/mL) and the mean plasma Cmax for RV (3.027 µg/mL) [24, 25] and the proposed models linearity and detection limits allowed this level of determination, were developed by transferring aliquots of different concentrations from FV and RV working solutions to a series of 10-mL centrifugation tubes together with 1 mL of human plasma and 3 mL of acetonitrile. The tubes were shaken for 1 min on a vortex mixer and centrifuged for 30 min. The formed supernatants were evaporated to dryness. The residues were dissolved in appropriate volume of ethanol, transferred to 10-mL volumetric flasks together with 3 mL of acetate buffer pH 4 and diluting to volume with ethanol. The samples were determined using the described procedures.

## Results and discussion

The integration and adaptation of chemometrics with spectrophotometric analysis represents a promising tool for achieving green chemical analysis. Since spectrophotometry is widely used in pharmaceutical quantitative analysis, researchers have developed many models for processing spectral data and enabling the determination of multiple components. In recent decades, CLS, PCR, and PLS models have been widely used for spectrophotometric analysis. In this work, the mentioned models were used for processing spectral data of FV and RV and allowed their determination in pharmaceutical form and spiked human plasma.

### Solvent choice regarding to green ranking

Recently, the choice of solvent is strongly recommended considering the health and environmental aspects of organic solvents. Solvent selection was based on the ranking of solvents with respect to green analytical chemistry metrics [26]. Our strategy was to reduce the impact of hazardous solvents and replace commonly used organic solvents such as acetonitrile and methanol, with greener ones. So far, ethanol was the appropriate alternative organic solvent.

### Models implementation for spectral data processing of FV and RV

**Fig. 2:**
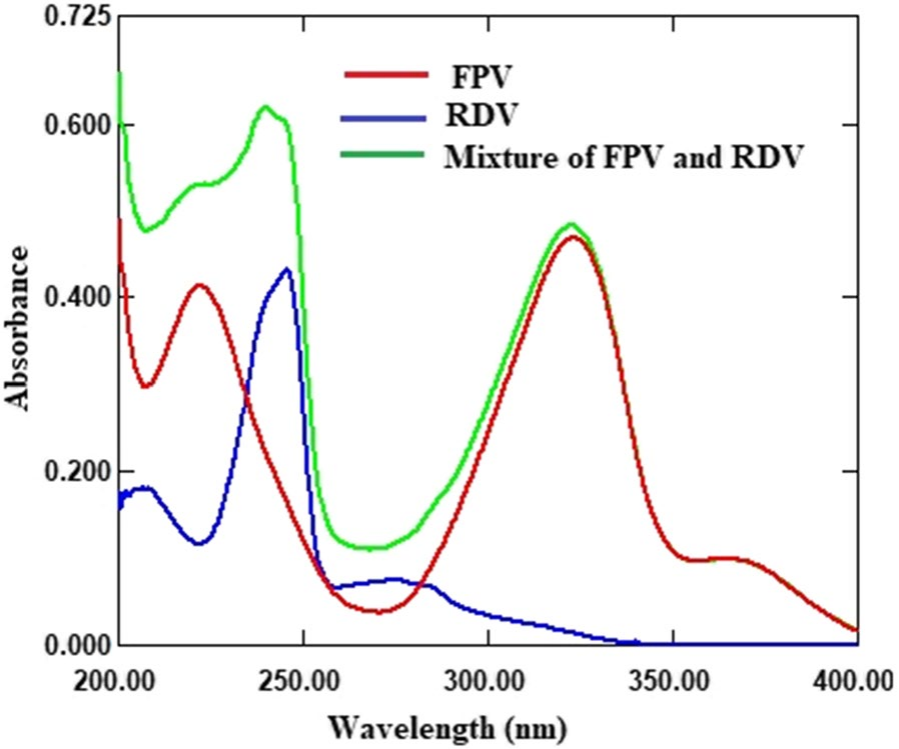
Absorption spectra of FV (9 µg/ mL), RV (9 µg/ mL) and their mixture (9 µg/ mL + 9 µg/ mL)

**Fig. 3:**
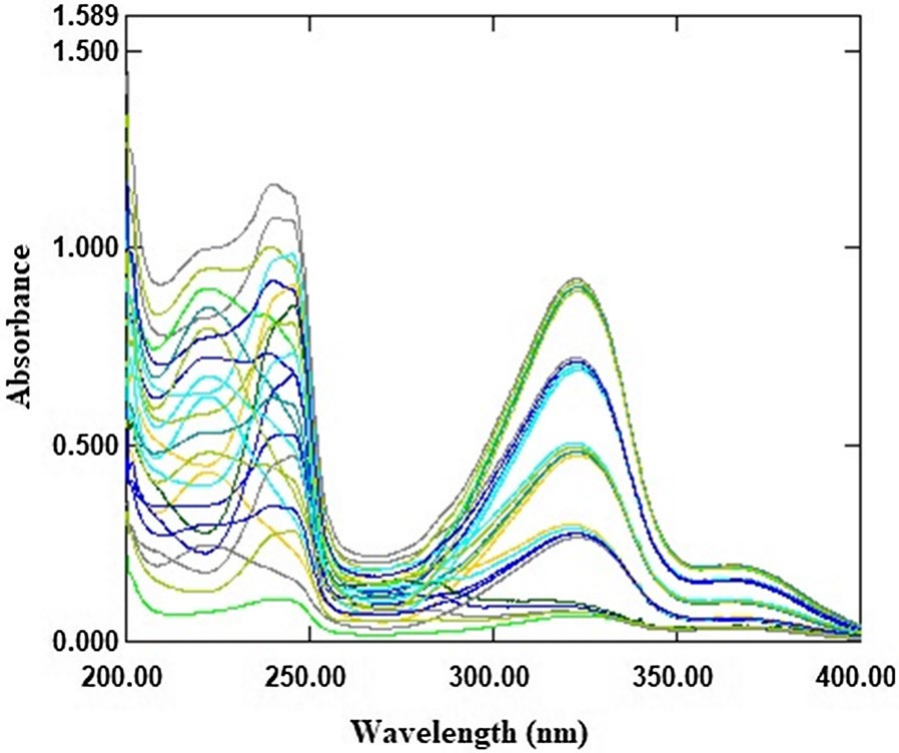
The absorption spectra of twenty-five mixtures of FV and RV planning mixtures

The UV absorption spectra of FV and RV, Fig. 2, overlap to some extent, making simultaneous determination difficult. To solve this problem, the described models were implemented. First, the assignment of calibration and validation sets was performed using the statistical design for generating the design sets of the recommended experiments. This step was based on the optimization approach with five-level, two-factor experimental design [27]. This optimization step provided organized defined mixtures of FV and RV. Twenty-five sample mixtures, Table 1, were prepared, spectrally scanned, Fig. 3, and divided into two groups. Thirteen samples were used to develop a calibration set. In addition, twelve samples were used to test the predictive power of the described models. When selecting drug concentrations for the model study, it was recommended to test the linearity range of FV and RV. The commercial concentration of the drugs should also be considered in the model construction. The spectral data of FV and RV were divided into two parts: Noise and Information. Noise was excluded because it represented non informative data. This data part was neglected because it had a negative impact on the significant data. The other spectral data, on the other hand, contained the relevant information of the samples studied.

**Table 1:** Five-level, two-factor experimental design developed to create the described models

Sample number	FV (µg/mL)	RD (µg/mL)
*1*	*9*	*9*
2	9	1
*3*	*1*	*1*
4	1	17
*5*	*17*	*5*
6	5	17
*7*	*17*	*9*
8	9	5
*9*	*5*	*5*
10	5	13
*11*	*13*	*17*
12	17	13
*13*	*13*	*9*
14	9	17
*15*	*17*	*17*
16	17	1
*17*	*1*	*13*
18	13	1
*19*	*1*	*9*
20	9	13
*21*	*13*	*13*
22	13	5
*23*	*5*	*1*
24	1	5
*25*	*5*	*9*

The italic values represent the calibration set

**Fig. 4:**
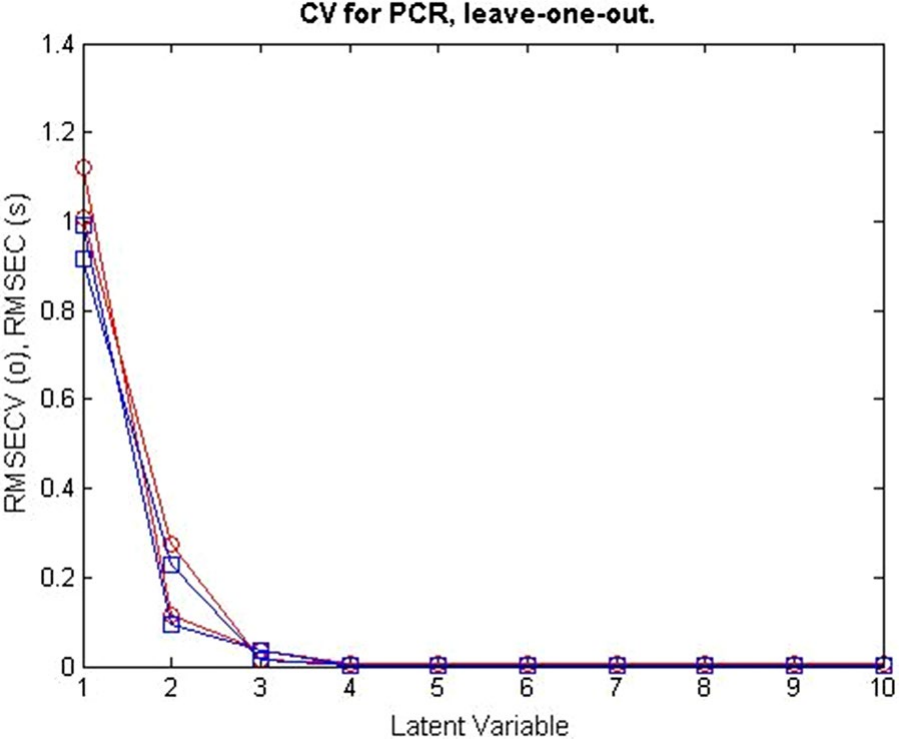
RMSECV plot of the calibration set results as a function of the number of LVs applied to develop the PCR model of FV and RV mixtures

**Fig. 5:**
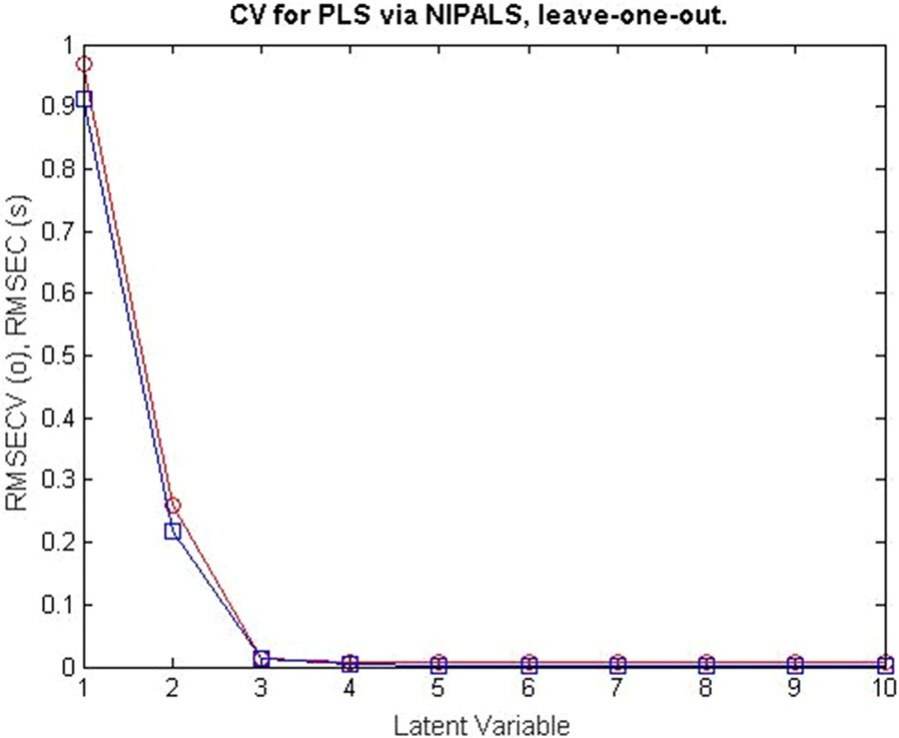
RMSECV plots of the calibration set results as a function of the number LVs applied to develop the PLS model of FV in FV and RV mixtures

**Fig. 6:**
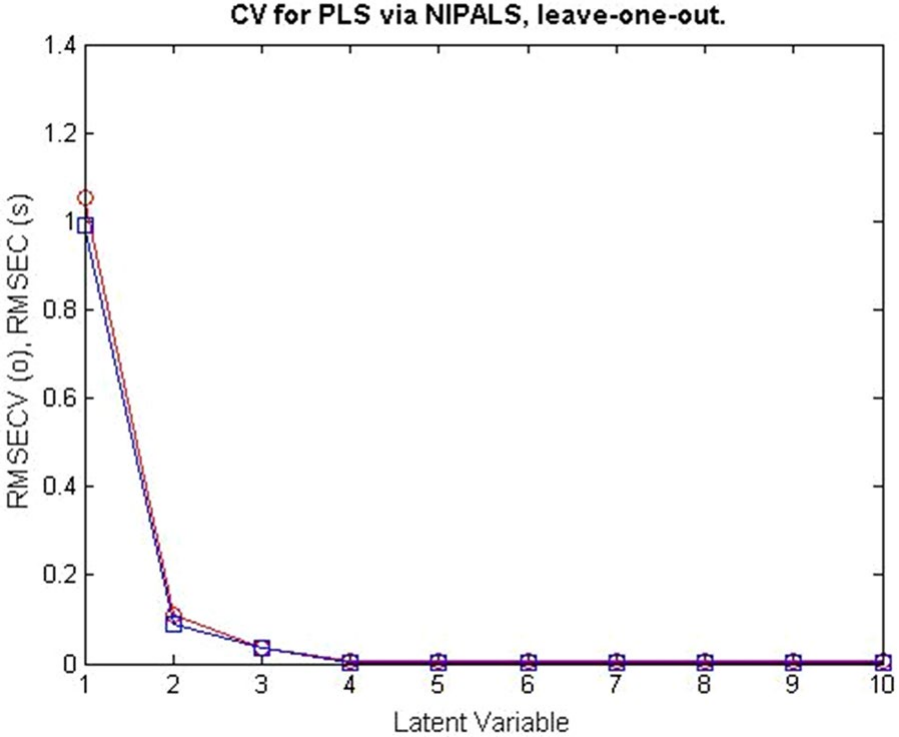
RMSECV plots of the calibration set results as a function of the number LVs applied to develop the PLS model of RV in FV and RV mixtures

CLS was the model described, which recommended that all compounds in calibration samples be detected. PCR, on the other hand, allowed the determination of the compounds FV and RV even in the presence of interfering compounds. This feature gave the PCR model the preference over the CLS model. Cross-validation was developed for PCR, omitting one sample at a time. The plot of the root mean square error of cross-validation [RMSECV] of the results of the calibration set as a function of the number of latent variables [LVs] used to develop the PCR model for the samples FV and RV samples was shown in Fig. 4. PLS offered many advantages over CLS and PCR. PLS provided analysts with complete information about the spectral data as well as the concentrations of the compounds under investigation. This improved the quality and predictive power of PLS. The RMSECV plots of the results of the calibration set as a function of the number of LVs used to build the PLS model for the samples of FV and RV are shown in Figs. 5, 6.

The evaluation and interpretation of the described model data was achieved. The evaluation of the obtained data and the statistical comparison with the accepted values were also considered. The percentage recoveries, the mean, the standard deviation [SD], the root mean square error of calibration [RMSEC] and the root mean square error of prediction [RMSEP] were listed in Tables 2 and 3.

**Table 2:** Results of the described models for the calibration set of FV and RV mixtures

Calibration set	CLS		PCR		PLS	
	FV	RV	FV	RV	FV	RV
1	97.83	100.88	100.03	100.09	100.03	100.00
2	99.70	99.07	99.65	99.09	99.66	100.00
3	101.48	99.53	100.05	100.22	100.05	100.00
4	98.38	100.86	100.06	100.14	100.06	99.99
5	101.67	99.56	100.07	100.13	100.07	100.02
6	101.66	99.57	100.01	100.11	100.01	100.04
7	98.08	100.87	99.99	100.12	99.99	99.99
8	101.66	99.56	100.07	100.13	100.07	100.02
9	101.85	99.51	100.01	99.98	100.01	99.97
10	97.01	100.90	99.99	100.04	99.99	100.01
11	101.59	99.49	100.00	100.06	100.00	99.95
12	99.92	99.06	99.77	99.12	99.78	99.99
13	97.54	100.89	100.08	100.07	100.08	100.01
Mean ± RSD	99.87 ± 1.855	99.98 ± 0.739	99.98 ± 0.131	99.93 ± 0.391	99.98 ± 0.125	100.00 ± 0.023
RMSEC	0.2191	0.0861	0.0137	0.0389	0.0136	0.0032

**Table 3:** Results of the described models for the validation set of FV and RV mixtures

Validation set	CLS		PCR		PLS	
	FV	RV	FV	RV	FV	RV
1	99.98	99.05	99.76	99.15	99.76	99.99
2	101.94	99.59	100.01	100.04	100.01	100.05
3	101.91	99.59	100.10	100.06	100.10	100.04
4	101.55	99.55	100.02	100.16	100.02	100.01
5	101.83	99.50	100.09	100.01	100.09	99.96
6	101.61	99.48	100.07	100.08	100.07	99.94
7	101.77	99.58	100.05	100.09	100.05	100.03
8	100.17	99.03	99.83	99.22	99.83	99.98
9	100.03	99.04	99.74	99.19	99.74	99.99
10	101.70	99.50	100.04	100.03	100.04	99.96
11	101.46	99.54	99.98	100.19	99.98	100.01
12	101.66	99.57	99.99	100.10	99.99	100.03
Mean ± RSD	101.30 ± 0.751	99.42 ± 0.234	99.97 ± 0.126	99.86 ± 0.409	99.97 ± 0.124	100.00 ± 0.035
RMSEP	0.1789	0.0717	0.0164	0.0419	0.0163	0.0046

### Methods validation

The models described was validated regarding to linearity range, accuracy, precision, limits of detection [LOD], limits of quantitation [LOQ] and selectivity parameters.

### Linearity range

Regarding to the developed experimental design which considered the range of 1–17 µg/mL for FV and RDV, accepted results were obtained over this concentration range as listed in Table 4.

**Table 4:** Performance and validation data of the described models for determination of FV and RV

Parameters	CLS		PCR		PLS	
	FV	RD	FV	RD	FV	RD
Wavelength scanning range	220–400	220–400	220–400	220–400	220–400	220–400
Linearity range (µg/mL)	1–17	1–17	1–17	1–17	1–17	1–17
LOD (µg/mL)	0.133	0.356	0.132	0.215	0.265	0.177
LOQ (µg/mL)	0.407	1.079	0.401	0.652	0.804	0.537
Accuracy (%R)^a^	100.79	100.94	99.78	99.69	98.77	100.23
Precision (%RSD)^b^						
Repeatability	0.934	0.870	0.870	0.721	0.658	0.524
Intermediate Precision	1.043	0.987	0.987	1.023	0.871	0.742

^a^Values for 9 determinations (3 concentrations repeated 3 times)

^b^Values for 9 determinations (3 concentrations repeated 3 times

### LOD and LOQ

LOD and LOQ were calculated based on the residual standard deviation of the considered linearity range (SD) and the slope using the following equations:

$$\text{LOD}=3.3\,\text{SD}/\text{slope}$$

$$\text{LOQ}=10\,\text{SD}/\text{slope}$$

The results obtained, Table 4, revealed the sensitivity of the described models.

### Accuracy

The accuracy of the developed models was monitored by calculating the mean percent recovery (%R), for triplicate determination of three concentration levels of each drug (4, 8, and 16 µg/mL). The accepted percent of recovery as listed in Table 4 demonstrated the high reliability of the developed models.

### Precision

The precision of the developed models was monitored by calculating the percent of relative standard deviation (%RSD), for triplicate determination of three concentration levels of each drug (4, 8, and 16 µg/mL) within one day for repeatability and on three successive days for Inter mediate precision. The small values of %RSD demonstrated high precision of the proposed models as listed in Table 4.

### Selectivity

The models selectivity was achieved by selective estimation of FV or RDV in the pharmaceutical form of each drug through application of standard addition technique. The results listed in Table 5 revealed that the models were sufficient selective for determination of the drugs under the study without interference from pharmaceutical excipients.

**Table 5:** Standard addition technique data for FV and RV using the described models

Method	Drug	Pharmaceutical taken ( µg/mL)	Pharmaceutical found (µg/mL)	Pure added (µg/mL)	Pure found (µg/mL)	% R
CLS	FV	2	1.99	3	3.02	100.67
				5	4.98	99.60
				7	7.09	101.29
	Mean± %RSD					100.52 ± 0.848
	RV	4	4.01	4	4.03	100.75
				6	5.95	99.17
				8	8.05	100.63
	Mean± %RSD					100.18 ± 0.880
PCR	FV	2	1.99	3	2.98	99.33
				5	5.05	101.00
				7	6.94	99.14
	Mean± %RSD					99.83 ± 1.023
	RV	4	3.98	4	3.95	98.75
				6	5.97	99.50
				8	7.87	98.38
	Mean± %RSD					98.88 ± 0.579
PLS	FV	2	2.00	3	3.02	100.67
				5	4.98	99.60
				7	6.96	99.43
	Mean± %RSD					99.89 ± 0.671
	RV	4	4.02	4	4.05	101.25
				6	6.08	101.33
				8	8.15	101.87
	Mean± %RSD					101.48 ± 0.334

### Application of the proposed models for determination of FV and RV pharmaceutical dosage forms

The described models were successfully applied to determine FV and RV in the pharmaceutical dosage forms. The results were statistically compared with those obtained by the reported method of FV [8] and RV [14]. By applying statistical tests, no significant differences were found at 95% confidence level, assuring the reliability of the described models for determination of FV and RV in the pharmaceutical dosage forms, Table 6.

**Table 6:** Determination of FV and RV in pharmaceutical tablets by described model and statistical comparison with previous reported methods

Parameters	CLS		PCR		PLS		Reported method [8]	Reported method [14]
	FV	RD	FV	RD	FV	RD	FV	RD
n^a^	5	5	5	5	5	5	5	5
Average (%Recovery)	99.59	100.36	99.63	99.49	100.17	99.21	100.05	100.45
%RSD	1.073	1.354	1.110	0.754	1.270	1.115	1.235	1.237
Student’s *t* test (2.306)^b^	0.615	0.104	0.555	1.471	0.151	1.668	**–**	**–**
*F value*(6.388)^b^	1.336	1.195	1.248	2.743	1.061	1.262	**–**	**–**

^a^Number of measurements

^b^The values in parenthesis are tabulated values of “*t* “and “*F*” at (P = 0.05)

### Determination of FV and RV in spiked human plasma

CLS, PCR and PLS models were successfully applied for the determination of FV and RV in spiked human plasma as they provided a high degree of linearity and detection which allowed the determination of FV, the mean plasma Cmax for FV (4.43 µg/mL) and RV, the mean plasma Cmax for RV (3.027 µg/mL) [24, 25]. The data in Table 7 showed that the described models were suitable for the determination of the studied drugs in human plasma without interference of endogenous components of the plasma matrix.

**Table 7:** Determination of FV, RV in spiked human plasma by the proposed models

Added (µg/mL)		%Recovery					
		CLS		PCR		PLS	
FV	RV	FV	RV	FV	RV	FV	RV
1	1	95.28	94.85	94.00	94.85	94.25	94.12
2	1	95.54	93.58	94.54	93.58	95.66	94.15
1	2	92.88	95.24	92.57	94.25	93.1	94.25
1	3	93.35	94.25	92.71	94.25	94.15	95.25
3	1	94.26	92.88	93.55	92.88	93.15	93.66
Mean ± RSD		94.26 ± 1.16	94.16 ± 0.95	93.47 ± 0.84	94.23 ± 0.75	94.06 ± 1.04	94.29 ± 0.58

### Green assessment of the described models

To evaluate and rank the greenness of the described models, analytical eco-scale score [21] was measured mainly depending on the amounts of solvents consumed. The analytical eco-scale is a semi quantitative metric considered that ideal green analysis has a value of 100. Penalty points were assigned for every analytical procedure parameter including amount of reagents, hazards, energy and waste, deviated from ideal green analytical chemistry principles. The penalty points of full procedure parameters were summed and subtracted from the basis of 100. It was reported that score more than 75 reveled excellent green analysis, score less than 50 score indicated inadequate green analysis, and score between 50 and 75 revealed acceptable green analysis. As the calculated scores were 79, 81 and 78 for CLS, PCR and PLS respectively these scores revealed the green excellence of the described models and had minimal negative impact on the environment and human health.

Furthermore, green analytical methods index introduces precise tool for view of different variables included in the analytical procedure. It considered sample preparation, sample handling (collection, preservation, transport and storage) as well as the chemicals used and the instrumentation. Every variable was colored from green to yellow to red which indicated either low, medium or high negative environmental impact, respectively [22]. The described models CLS, PCR, PLS had the 5 green zones and one red zone.

Finally, the analytical greenness measure (AGREE) tool was used, which presents the environmental friendliness profile of the analytical methods as a numerical value. AGREE is a user-friendly software that uses the twelve-significance principle of green analytical chemistry as input criterion. Each of the twelve inputs was scored according to range from 0 to 1 and mirrored on the intuitive red-yellow-green color scale. The weight of every input criterion was assigned according to its role in the procedure, and this was reflected by the width of its corresponding segment. The output was a clock-like graph, with an overall score and color representation in the middle [23]. The obtained ultimate score was 0.82, 0.84, 0.80 for CLS, PCR and PLS respectively. These results confirmed the superior green characters of the developed models. AGREE tool takes into account the quantities of any reagent used. It is easy to construct and shows the weaknesses of the studied method. In summary, the results of the green metrics presented a detailed environmental friendliness profile and confirmed compliance with environmentally friendly practices in most cases [28–34]. The green assessment results were presented in Table 8.

**Table 8:** Green assessment results of the proposed chemomertric models

Parameter	CLS	PCR	PLS
National environmental method index			
Green analytical procedure index			
The AGREE evaluation method			

### Advantages of the models described over the previous reported methods

The present work demonstrated the feasibility of green fitted CLS, PCR and PLS chemometric based models for simultaneous spectroscopic determination of FV and RV in the pure form, pharmaceutical dosage and spiked human plasma. They were applied in the spectra data evaluation providing the synchronous inclusion of many wavelengths rather than using a single wavelength. This greatly increases the precision ability of the analysis process. The described models provided some advantages compared to the conventional methods as they were non-destructive and performed directly to samples without any extraction procedures, unnecessary performing stability studies. and cost-effective. Moreover, the analysis time of the developed models was shorter and the solvent consumption was much lower than previous reported works.

## Conclusion

Adjusted green fitted spectrophotometric models, classical least squares, principal component regression, and partial least squares were developed for the determination of favipiravir and remdesivir in pharmaceutical form and spiked human plasma. The chemometric models were constructed and optimized for integrated green spectrophotometric determination of the studied drugs. Greenness evaluation of the described models was done using, the analytical eco-scale, the green analytical procedure index and the AGREE evaluation method. The results showed that the described models complied and met the environmental friendliness characteristics in terms of the official green metric values. The results confirmed the minimal impact of the developed models on the environment and the general health of the analysts. The models offered successful feasibility for the spectrophotometric determination of favipiravir and remdesivir in spiked human plasma.

## Data Availability

The datasets used and/or analyzed during the current study are available from the corresponding author on reasonable request.

## Abbreviations

FV: Favipiravir
RV: Remdesivir
CLS: Classical least squares
PCR: Principal component regression
PLS: Partial least squares
